# Supplementary material for: Identification of photoperiod-induced specific miRNAs in the adrenal glands of Sunite sheep (Ovis aries)
Source: Front Vet Sci. 2022 Jul 22;9:888207. doi: 10.3389/fvets.2022.888207 (PMC9354845; doi:10.3389/fvets.2022.888207)
Supplement: Supplementary file 1 [file Data_Sheet_1.ZIP › Supplementary file/miRDeep2 file.zip!/miRDeep2 file/Know_Structure/LP42A3/oar-mir-758.pdf]

miRBase precursor : oar-mir-758
Total read count : 1022
oar-miR-758-5p read count : 0
oar-miR-758-3p read count : 1015
remaining reads : 7

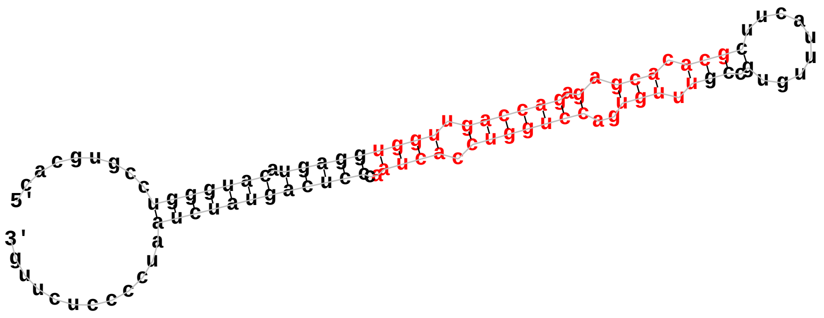

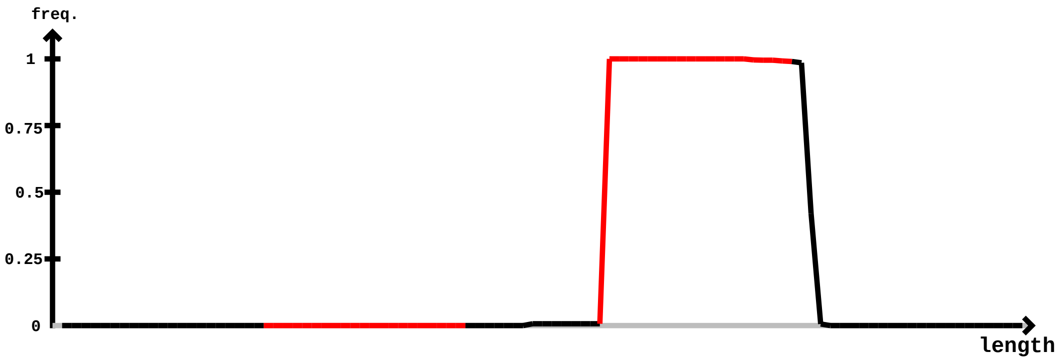


```
                                                           oar-miR-758-3p
                             oar-miR-758-5p
5'- cacgugccuggguacaugaggugguugaccagagagcacacgcuucauuugugccguuugugaccugguccacuaacccucaguaucuaauccccucuug   -3'    exp
    ........((((((.(((((((((.((((((.(.(((.((((.........).))).)))..))))))).)))).))))))))))))...........      reads    mm    sample
    ................................................uugugccguuugugaccuggucc.............................      4        0     seq
    ................................................uugugccguuugugaccuggucca............................      1        0     seq
    ................................................uugugccguuugugaccugguccacu..........................      2        0     seq
    ........................................................uuugugaccugguccacu..........................      1        0     seq
    ........................................................uuugugaccugguccacua.........................      2        0     seq
    ........................................................uuugugaccugguccacuaa........................      5        0     seq
    ........................................................uuugugaccugguccacuaac.......................      578      0     seq
    ........................................................uuugugaccugguccacuaacc......................      423      0     seq
    ........................................................uuugugaccugguccacuaaccc.....................      6        0     seq
```
